# Bi-Ligand Modification of Nanoparticles: An Effective Tool for Surface-Enhanced Raman Spectrometry in Salinated Environments

**DOI:** 10.3390/nano9091259

**Published:** 2019-09-05

**Authors:** Anna Tycova, Karel Kleparnik, Frantisek Foret

**Affiliations:** 1 Institute of Analytical Chemistry of the Czech Academy of Sciences, Veveri 967/97, 602 00 Brno, Czech Republic (A.T.) (K.K.); 2 Central European Institute of Technology, Masaryk University, Kamenice 753-5, 602 00 Brno, Czech Republic

**Keywords:** ionic strength, nanoparticles, modification, myoglobin, saline solution, SERS, silver, sintering, stability, surface-enhanced Raman spectrometry

## Abstract

Elimination of massive aggregation of nanoparticles in the sample of high ionic strength is a prerequisite for the sensitive analysis through a surface-enhanced Raman spectrometry (SERS). We present a system of silver colloid modification composed of two thiolated modifiers (3-mercaptopropionic acid and thiolated polyethylene glycol) both creating a strong Ag-S bond. At their optimal molar ratio, the polymer acts as a steric barrier preventing direct nanoparticle–nanoparticle interaction, while the low-molecular organic acid creates areas accessible for the analyte molecules. Thus, this approach is an excellent tool for sustaining both the colloidal stability and SERS sensitivity. The functionality of the system was demonstrated on the SERS analysis of myoglobin from a saline solution. The favorable creation of hot spots was achieved by laser-induced sintering.

## 1. Introduction

Surface-enhanced Raman spectrometry (SERS) combines the potential of Raman spectrometry for a definite identification of an analyte with remarkable sensitivity achieved by the surface enhancement effect occurring on metal nanoparticles [1]. The surface chemistry of the colloid is a crucial characteristic influencing its biocompatibility, affinity, or stability [2,3,4,5]. Indeed, after its synthesis, a colloid is usually stabilized by repulsive coulombic forces between the nanoparticles occurring as a result of the ion layer of a stabilizing agent [6,7]. At an increased ionic strength, the repulsive forces weaken, and the nanoparticles (NPs) form larger entities.

Since the surface enhancement effect employed in SERS can occur only on nanostructures, the uncontrolled aggregation process can severely influence the sensitivity of the measurement. Pamies et al. evaluated the ionic strength of 0.1 M as a critical level for the massive aggregation process of gold colloid [8]. Many real-world samples (e.g., biological fluids, food extracts, and some environmental samples) exceed this level easily. Thus, the stabilization of colloids in salinated samples is essential [9]. Unfortunately, commonly used polymeric modifiers act as a barrier shielding an analyte from the electromagnetic field at the nanoparticle surface [10,11]. Therefore, the typical strategy for an SERS measurement of samples of high ionic strength lies in the application of expensive solid substrates or other complicated protocols [12].

In this work, we introduce a unique system of surface modification of silver nanoparticles composed of two types of ligands—thiolated polyethylene glycol (PEG) and 3-mercaptopropionic acid (MPA). Whereas the polymer acts as a steric barrier for the NP–NP interaction preventing their aggregation, a short molecule of MPA allows the analytes to get close to the nanoparticle surface and take advantage of the surface enhancement. This simple original modification opens a way to fast and straightforward SERS measurements of real-world samples without any need for complicated equipment or protocol.

## 2. Materials and Methods

The silver colloid was synthesized by a standard Lee-Meisel protocol based on the reduction of silver nitrate (Sigma Aldrich, St. Louis, MO, USA) by a sodium citrate dihydrate (Lachema, Brno, Czech Republic) [6]. Into 150 mL of boiling deionized water, 3 mL of 55 mM AgNO_3_ and 4.8 mL of 20 mM sodium citrate dihydrate were added. The mixture was boiled for 90 min using a reflux cooler. Typically, the obtained colloid provided relatively a broad size distribution with an average size of particles in the range of 80–90 nm and with standard deviations of 30–40 nm. Figure S1 shows the TEM figures of the colloid.

**Figure 1:**
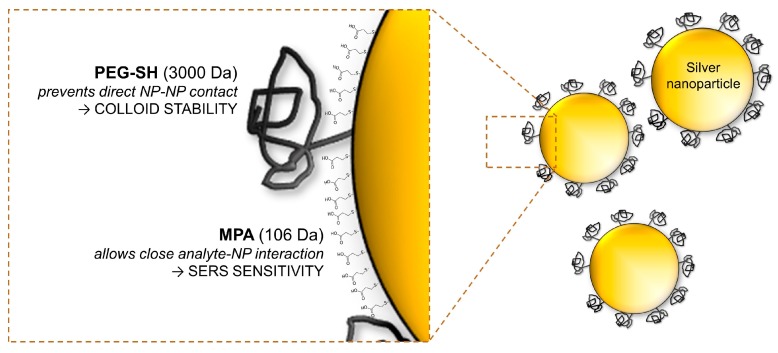
The bi-ligand design of the nanoparticles (NPs) surface modification for sensitive surface-enhanced Raman spectrometry (SERS) measurements using a combination of the thiolated polyethylene glycol (PEG) and 3-mercaptopropionic acid (MPA). The figure elements are not in the scale.

For its surface modification (Figure 1), the mixture of the 3-mercaptopropionic acid and *O*-(3-Carboxypropyl)-*O*′-[2-(3-mercaptopropionylamino)ethyl]-polyethylene glycol (3000 Da) were used. These compounds were mixed in deionized water in the molar ratio of 10:1 (MPA:PEG) to achieve their concentration of 20 µM (see Table S1). This reaction mixture was added into a silver colloid in the ratio of 1:1 (*v*/*v*) and immediately vortexed for 2 min, followed by mild mixing for the next 30 min. Myoglobin (from the bovine heart) was dissolved in 1.8% (*v*/*w*) of NaCl and introduced into the modified colloid in the ratio of 1:1 (*v*/*v*), thus, obtaining the concentration of a saline solution. The SERS measurements were conducted within 5 min after the sample preparation. If not stated otherwise, the chemicals were purchased in Sigma Aldrich (St. Louis, MO, USA).

The colloids were characterized by a UV-Vis spectrometer (UV-1800, Shimadzu, Kyoto, Japan) with a spectral resolution of 1 nm, and dynamic light scattering (ZetaSizer Nano ZS, Malvern Instruments, Malvern, UK). A laboratory-constructed Raman system based on the epifluorescence microscope body (JENALUMAR, Carl Zeiss Jena, Jena, Germany) consisted of a 50× microscope objective, He-Ne laser (633 nm, 15 mW, Melles Griot, Rochester, NY, USA), Czerny-Turner spectrograph (Shamrock SR-303i, Andor, Belfast, UK) and deep cooled back-illuminated CCD detector (iDus, Andor, Belfast, UK) [13]. The system provided a spectral resolution of 3 cm^−1^ and the laser spot diameter of 20 µm. Every sample was measured seven times. For experiments with myoglobin, the nanoparticles were concentrated by centrifugation to 3 mg/mL. The signal was collected from the sample droplets formed on hydrophilic spots within a hydrophobic platform (see Figure S2). During the SERS measurements, the silver nanoparticles immediately sintered forming a large elongate aggregate.

## 3. Results and Discussion

In SERS, the effect of the surface enhancement is particularly strong in so-called hot spots—i.e., places of closely located nanostructures. To induce the formation of hot spots in the colloids, a mild increase of ionic strength is usually employed. However, in many real-world samples, the high ionic strength makes the process of aggregation extremely fast. This ruins their colloidal character and thus, the potential for the enhancement effect [8].

We modified the surface of the colloid by two ligands (Figure 1) of very different properties: (1) PEG—a neutral polymer acting as a steric barrier for NP–NP interactions and (2) MPA—a short organic acid creating negatively charged areas accessible for an analyte. Both of the compounds contain a thiol functional group interacting efficiently with the silver surface by the formation of an Ag–S covalent bond.

The complete saturation of the colloid surface plays a key role in its stability. The optimum molar concentration of MPA entering the coating reaction was determined by following the changes in the absorption maxima shown in Figure S3. The consecutive saturation of the colloid causes its shift from 420 nm to 424 nm. This way, we determined the concentration of 20 µM of the modifier as the most appropriate.

**Figure 2:**
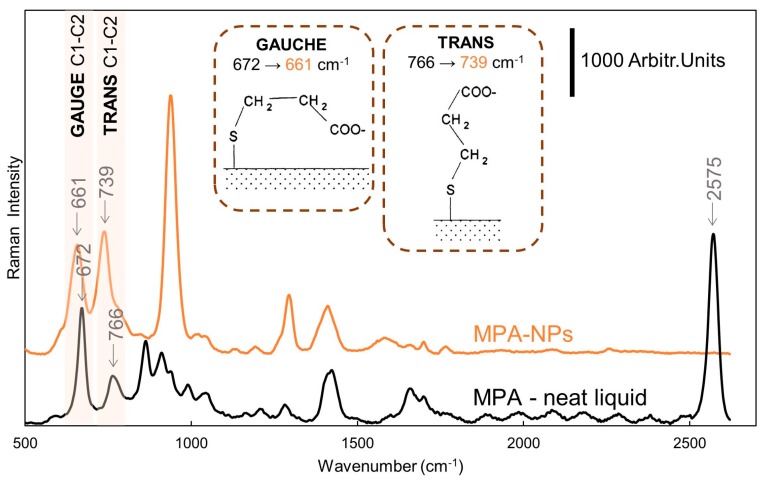
Comparison of the Raman spectrum of the MPA (neat liquid) and SERS spectrum of nanoparticles modified by MPA (0.5 mg/mL). The accumulation time was 3 × 15 s and 3 × 5 s, respectively. For a better visual comparison of the spectra, an offset, background correction, and smoothing was used.

The binding of MPA on the nanoparticle surface was confirmed by Raman/SERS measurements too. Figure 2 compares the Raman spectrum of MPA with the SERS spectrum of NPs modified by MPA. The Raman spectrum of the pure MPA is dominated by an intensive band at 2575 cm^−1^, which corresponds to the S-H vibration. This band was not observed in the SERS spectrum of MPA–NPs, suggesting the binding of a thiol group to the silver. Another important information is the shift of the bands located at 672 cm^−1^ and 766 cm^−1^ to 661 cm^−1^ and 739 cm^−1^, respectively, which is another well-documented proof of the MPA–Ag interaction [14,15]. These bands are assigned to C1–C2 vibrations of gauche and trans conformations of the MPA molecule. Notably, the increase in the ratio of peak intensities at 739 cm^−1^ and 661 cm^−1^, in the MPA–NPs spectrum, with respect to the ratio of intensities at 766 cm^−1^ and 672 cm^−1^, in the neat MPA spectrum, indicates the formation of a dense MPA layer with a prevailing trans conformation. The spectrum of MPA–NPs is dominated by 935 cm^−1^, 1294 cm^−1^, and 1415 cm^−1^ assigned to υ(C-COO), υ(CO)+ δ(OH), and υ(CO) + δ(OH)+, respectively. The detailed assignment of all the MPA–NPs and MPA bands is nicely overviewed elsewhere [15]. Interestingly, since the MPA fully saturated the Ag–NPs surface (thus replacing stabilizing citrates), no interferences by the citrate molecules were recognized in the SERS spectrum.

**Figure 3:**
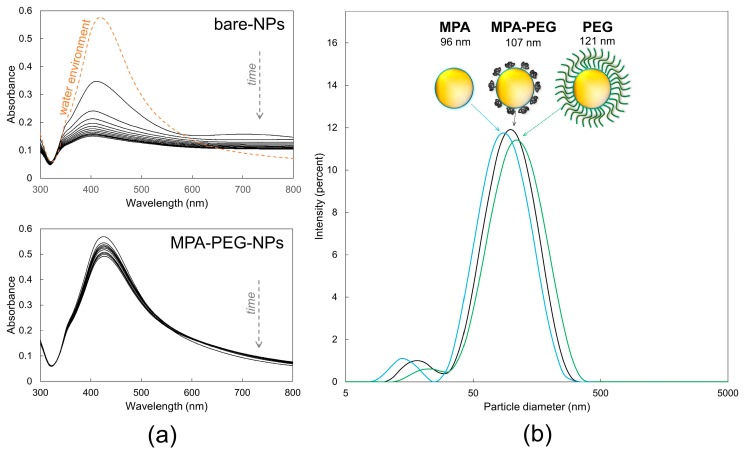
(**a**) The stability measurements of colloids (10 µg/mL) in a saline solution within 60 min. The spectra were collected every 5 min; (**b**) dynamic light scattering (DLS) measurements of the influence of PEG conformation on the particle size.

The high surface density of the polymer would create a dense layer preventing an analyte to get to the nanoparticle surface, which is a crucial aspect for the sensitive SERS analysis. Therefore, we searched for a minimal concentration of PEG in the reaction mixture, forming a steric barrier reliably sustaining a stable dispersion in a saline solution. This was fulfilled for a molar ratio of 10:1 (MPA:PEG). Since the system instability can be easily revealed by a UV-Vis absorbance [8], the absorption spectra of bare-NPs and MPA–PEG–NPs were compared in the environment of saline solution in the time scale of 60 min. Figure 3a shows the decrease of the absorbance of bare-NPs and MPA–PEG–NPs in time as a consequence of nanoparticle aggregation (i.e., shifts in the surface plasmon resonance). The bare colloid underwent immediate changes forming larger structures, while the modified colloid showed only a minimal drop. By testing five various batches of modified colloids, we observed the drop, which was always below 12%.

The conformation of PEG is influenced by its surface density. While at a high level of surface saturation this polymer creates a so-called *brush*, the desired *mushroom-like* areas, which do not block the surface of NPs, are formed only at the low saturation [16]. Using dynamic light scattering we investigated the nanoparticle size of three colloids treated by various modification protocols. If the surface of the colloid was saturated with either MPA or PEG only, their average diameter was 96 and 121 nm, respectively. The reaction mixture of MPA:PEG (10:1) formed stable NPs with a diameter of 107 nm (Figure 3b). This indicates that the molar ratio of 10:1 resulted in the formation of the mushroom conformation with an estimated length of about 5.5 nm.

Although the presented surface modification eliminates the massive uncontrolled aggregation, it also suppresses a mild level of aggregation, which positively influences the SERS signal due to the hot spots formation. Thus, during our SERS experiments, laser-induced sintering was employed to obtain *hot spots* in the probed area. The sintering of nanoparticles is a natural phenomenon occurring as a result of local overheating and a high concentration of nanoparticles [13,17,18].

**Figure 4:**
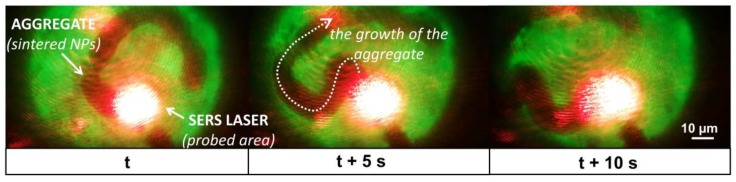
Microscopic observations of the development of a laser-induced aggregate within 10 s.

To achieve this, the nanoparticles were centrifuged to increase their concentration to 3 mg/mL in the sample. For the sintering of the colloid, the laser was focused on the sample droplet on the SERS platform (see Figure S2), where the sintering of nanoparticles occurred approximately after 2–5 s after the beginning of the laser exposure. The energy of the laser caused slight heating of the solvent resulting in a mild flow of the sample. This drift caused a continuous generation of the aggregate of an elongated shape with an approximate width of 5–15 µm. The average speed of the growing is estimated at 2 µm/s. Figure 4 shows the microscopic observations of the sample during the process of the nanoparticle sintering within 10 s. However, the continuous generation of the aggregate did not significantly influence the signal stability, and the monitored variances correspond to values typical for the SERS experiments (Figure S4b).

**Figure 5:**
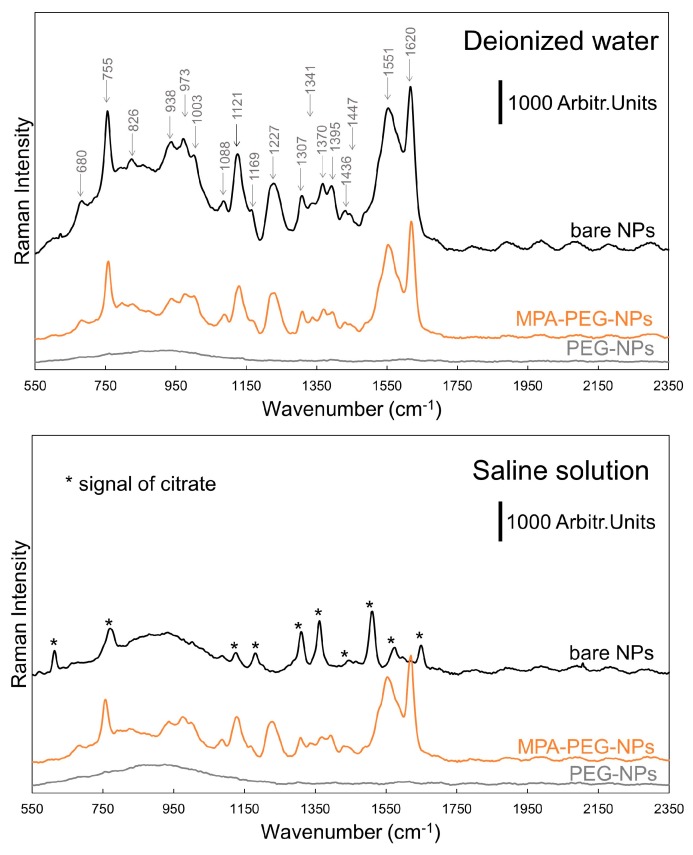
The typical SERS spectra of myoglobin (50 µM) using NPs of various surface modifications in a deionized water and saline solution. Exposure time: 3 × 10 s. Concentration of NPs: 3 mg/mL. For a better visual comparison of the spectra, an offset, background correction, and smoothing was used.

The performance of designed nanoparticles was tested in the SERS experiments using myoglobin. Figure 5 compares the SERS performance of unmodified NPs, MPA-PEG-NPs, and PEG-NPs in a deionized water and saline solution.

Based on our DLS measurements, the PEG polymer creates a coating of the approximate thickness of 12.5 nm, thus shielding the analyte molecules from the most intense electromagnetic field occurring on the nanoparticle surface. Due to this fact, no significant bands were monitored in both tested environments for the PEG-NPs.

On the other hand, bare nanoparticles provided an excellent myoglobin signal in deionized water with the character being in the perfect agreement with the previous studies [19,20]. However, in a saline solution nanoparticles massively aggregated, which significantly suppressed their potential for the enhancement effect and only citrate bands were detected. The citrate acts as a stabilizing agent of the colloid (see the details about its synthesis in Materials and Methods). Moreover, we deduce, that the process of aggregation is faster than the sorption of myoglobin on the nanoparticle surface. Thus, most of the analyte stayed outside of the aggregate without a minimal chance to get into the hypothetically formed *hot spots* inside the aggregate.

The MPA–NPs showed a very similar pattern of behavior (Figure S5b). Despite the strong SERS signal of the MPA bound on the NPs, the myoglobin spectrum was still apparent in the region of 1100–1700 cm^−1^. However, in a saline solution, the analyte response was completely covered by the MPA bands.

The combination of PEG and MPA created a stable system, sustaining a nanostructured character and providing sufficient time scale for the analyte-NPs interaction, resulting in well-resolved spectra in both tested environments. This proves its potential for being a useful tool in the SERS experiments in highly salinated samples. It should be stressed, that even though the MPA creates in the presented system the areas with improved accessibility for the analyte, it still mildly shields the analyte from the electromagnetic field around the nanostructures. On the other hand, we believe that the choice of a short-chained ligand can significantly influence the selectivity of analyses [21,22,23]. The blank spectrum of the MPA–PEG–NPs is shown in Figure S5a.

## 4. Conclusions

Nanoparticles represent a feasible tool for surface enhancement in the SERS experiments. However, in some cases, the stability limits their application. Therein, we have introduced a promising bi-ligand strategy of surface modification for improved stability at a sustained SERS performance. The essence of the coating lies in the combination of a steric barrier (a polymer) and accessible locations (a short organic acid). We proved that this strategy reliably allows analyzing myoglobin from the environment of a saline solution, while the untreated silver colloid provided only background noise. Since the presented surface modification prevents the mild level of aggregation positively influencing the SERS sensitivity, we induced hot spots by laser-induced sintering continuously forming elongated aggregates boosting the sensitivity of our measurements.

## Supplementary Materials

The following are available online at https://www.mdpi.com/2079-4991/9/9/1259/s1, Figure S1: TEM figures of the colloid synthetized by Lee-Meisel protocol, Table S1: The volumes of reagents used for surface modification of silver colloids, Figure S2: Design of hydrophobic platform with the hydrophilic spots, Figure S3: The absorption curves of colloids modified by various concentrations of MPA, Figure S4: The overall view on sample spots and stability of SERS signal, Figure S5: The performance of MPA–NPs and blank signal of MPA-PEG-NPs in SERS experiments.
