# Supplementary material for: Bi-Ligand Modification of Nanoparticles: An Effective Tool for Surface-Enhanced Raman Spectrometry in Salinated Environments
Source: Nanomaterials (Basel). 2019 Sep 5;9(9):1259. doi: 10.3390/nano9091259 (PMC6781045; doi:10.3390/nano9091259)
Supplement: Supplementary file 1 [file nanomaterials-09-01259-s001.pdf]

Support information

Bi-Ligand Modification of Nanoparticles: An Effective Tool for Surface-Enhanced Raman Spectrometry in Salinated Environments

Anna Tycova[1], Karel Kleparnik[1], Frantisek Foret[1,2]

[1] Institute of Analytical Chemistry of the Czech Academy of Sciences, Veveri 967/97, 602 00 Brno, Czech Republic
[2] Central European Institute of Technology, Masaryk University, Kamenice 753-5, 602 00 Brno, Czech Republic

Characterization of the silver colloid by transmission electron microscopy

The colloid synthetized by Lee-Meisel protocol was characterized by transmission electron microscopy (FEI Morgagni 268D, Thermo Fisher Scientific, Waltham, MA, USA). Figure S1 shows relatively high polydispersity being a typical characteristic of this kind of colloid.

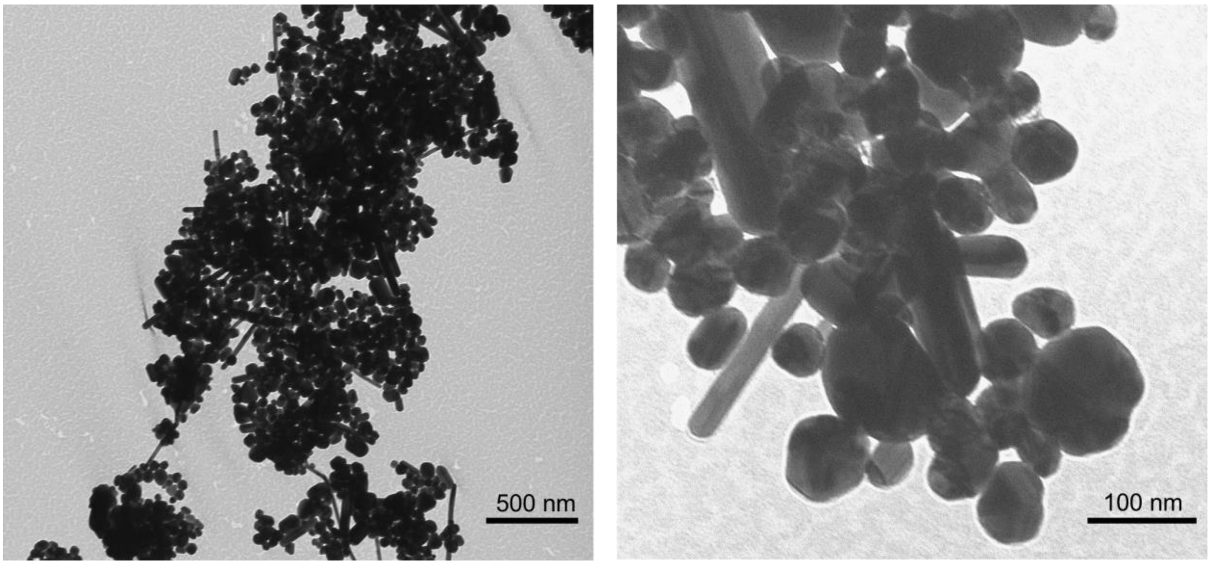

Figure S1. TEM figures of the colloid synthetized by Lee-Meisel protocol.

Protocol for the surface modification of colloids

In this work, several surface modifications were tested. Table S1 summarizes the additions of the reagents used for the modification protocols. In the first step, MPA and/or PEG were added into the deionized water and vortexed properly. Then, the solution was added to the colloid and vortexed for 2 minutes followed by further mild mixing for the next 30 minutes.

Table S1. The volumes of reagents used for surface modification of silver colloids.

Surface modification	Reagents			
	**MPA** **(1 mM)**	**PEG** **(10 mM)**	**deionized water**	**colloid**
NPs	x	x	1000 µL	1000 µL
MPA-NPs	40 µL	x	9960 µL	1000 µL
PEG-NPs	x	4 µL	9960 µL	1000 µL
MPA-PEG-NPs	36 µL	4 µL	9960 µL	1000 µL

Fabrication of the platform for SERS measurements

A platform for SERS experiments was fabricated from a microscopic glass slide. After plasma activation, the glass was exposed for 5 min to vapors of trichloro(1H,1H,2H,2H-perfluorooctyl)silane (Sigma Aldrich, St. Louis, MO, USA) and baked on a hot-plate (10 min, 110 °C) achieving stable and strongly hydrophobic surface. This surface was removed in several places via sandblasting using a metal mask with holes of 1 mm diameter. The diameter of the sand (Uni-max, Uzice, Czech Republic) was within the range of 20–60 µm and was introduced to the glass surface at a pressure of 2.4 bar. This way, a hydrophilic array of 91 spots was created particularly suited for SERS analysis of polar (i.e., water-based) samples (Figure S2). The whole fabrication process did not take more than 20 min. The platform was designed for analysis of 12 samples in 7 repetitions.

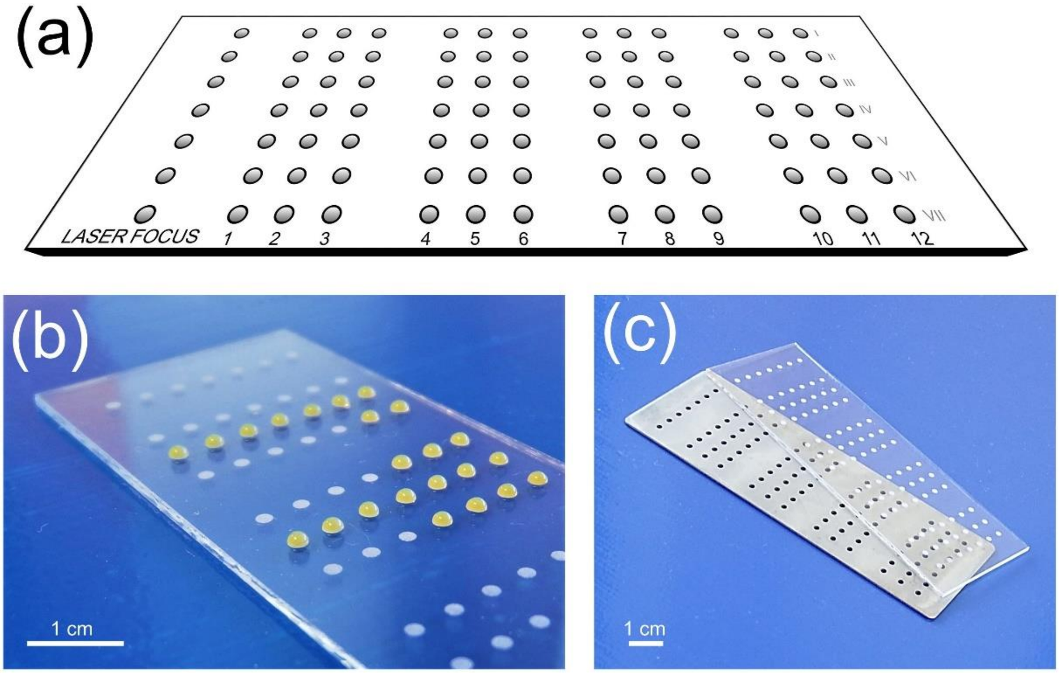

Figure S2. (**a**) Scheme of a hydrophobic platform with the hydrophilic spots; (**b**) The real design with several drops of the sample; (**c**) The overall view on the platform with a metal mask used for sandblasting.

Optimization of the concentration of the modifier

The complete saturation of the colloid surface plays a key role in its stability. Since surface plasmon is influenced by the surface chemistry of the nanoparticles [1,2], the suitable molar concentration of MPA was investigated by following the absorption maxima in UV-Vis spectra. The saturation of the colloid causes its shift from 420 nm to 424 nm, which was reached at the level of 10 µM (Figure S3). To cover possible batch-to-batch variances between the synthetized colloids, we determined the concentration of 20 µM as the most appropriate.

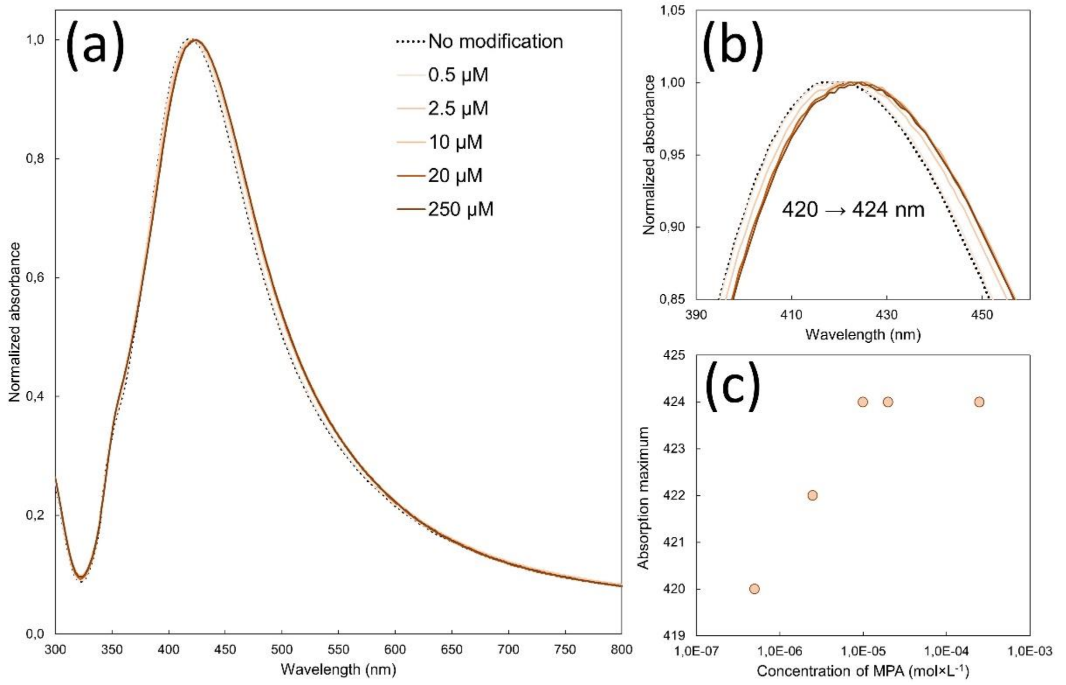

Figure S3. (**a**) The absorption curves of colloids modified by various concentrations of 3-mercaptopropionic acid; (**b**) A detail of the absorption maxima; (**c**) The shift plotted as the dependence of the absorption maximum on the MPA concentration.

Laser-induced sintering

Sintering is a process of making a compact material after the application of external energy (typically heat or pressure) without the liquefaction of the minor components. In this work, we employed laser-induced sintering to obtain favorable aggregation of stabilized nanoparticles resulting in an increased amount of *hot spots* in the probed area. Due to the thermal heating of solvent, the formed aggregate drifted in the sample droplet creating an elongated shape. The example of an appearance of the analyzed spot after the measurement is in Figure S4a.

The continuous generation of the aggregate did not significantly influence the signal stability. Figure S4b compares the SERS response of myoglobin (following band 1622 cm^{-1}) in five various samples within 80 s. In most of these measurements, the RSD of the signal was at the level of 20%, which corresponds to values typical for SERS experiments.

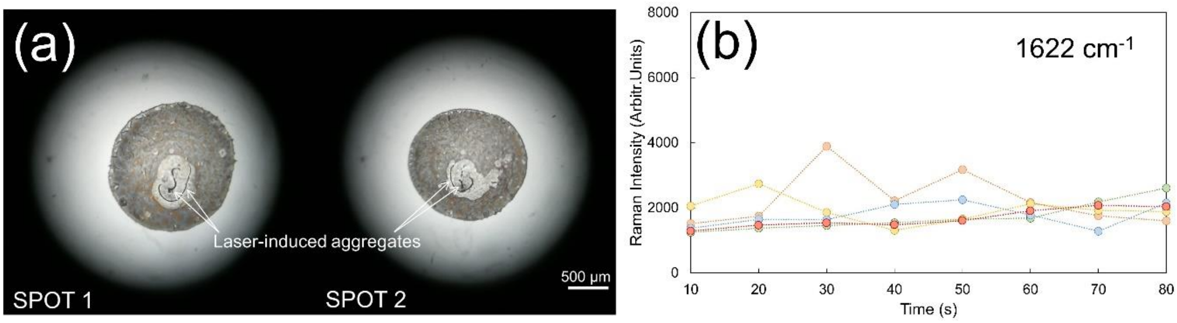

Figure S4. (**a**) Example of two sample spots after SERS measurement with laser-induced aggregation; (**b**) Stability of SERS signal (n = 5) during the analysis of 25 µM myoglobin, exposure time: 1 × 10 s.

SERS measurements of myoglobin

Modification of silver nanoparticles by MPA, PEG, MPA–PEG (10:1) was in this work compared to the performance of bare (untreated) nanoparticles in the environment of deionized water and saline solution. Myoglobin was chosen as a model molecule. Coating by thiolated PEG completely

ruined the SERS sensitivity in both tested environments due to the bulky dimension of the coating. In deionized water, MPA–NPs provided relatively nice myoglobin spectrum in the region of 1100–1700 cm^{-1}; however, at lower frequencies, the signal was completely devastated by the signal of MPA itself. Moreover, the stabilization by MPA is not very effective and in saline solution, the massive aggregation resulted in the signal of MPA only (Figure S5b).

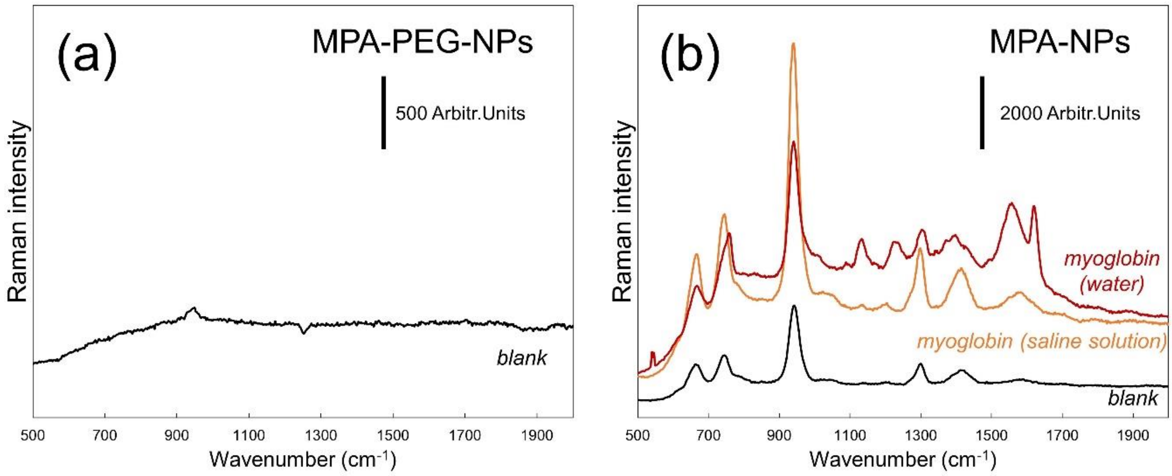

Figure S5. (**a**) The blank SERS spectrum of MPA–PEG–NPs in the water environment. (**b**) The performance of MPA–NPs in SERS experiments for analysis of myoglobin in saline solution and water environment, respectively. The experimental conditions are equal to those in Figure 5.

Thus, the combination of MPA and PEG in the molar ratio of 10:1 was evaluated as the most potential. It provided well-resolved spectra in water as well as in saline solution without significant background interferences (Figure S5a) suggesting that PEG influenced the enhancement of MPA signal or dramatically decrease the amount of its concentration on the nanoparticle surface. However, the direct explanation of this minimal background signal is not very clear.

One can deduce five possible explanations: (1) The PEG molecules act as a spacer preventing effective employment of the enhancement effect (by keeping NPs in the long distances). However, from our DLS experiments, the length of PEG is estimated at 5.5 nm, which still should result in relatively strong signal enhancement. Moreover, no myoglobin signal would be monitored if PEG played the role of the spacer. (2) PEG structures adhere to the nanoparticle surface instead of forming mushroom-like structures. This would minimize the area for MPA–NPs binding. This hypothesis would be against well-investigated structural behavior of PEG [3,4] and our DLS measurements. Further, we assume, that the reaction kinetics of the large PEG will be slower than the one of small organic acid. Thus, MPA should interact with NPs more easily than the polymer. (3) The presence of PEG in the reaction mixture results in the slight pH shift, influencing dissociation of MPA, thus, its SERS spectrum. Castro *et al.* investigated MPA–NPs response at two various pH values – i.e., pH = 5 and pH = 10 [5]. Both reported pH values represent relatively extreme conditions; however, the obtained SERS spectra differ only in a minimal way. (4) The binding of PEG on the nanoparticle surface influences the electron density of MPA molecule. PEG represents oxygen-rich structure (i.e., system of high electronegativity), which could theoretically change the electron density within MPA, thus reducing the polarizability and its SERS response. (5) Finally, PEG molecules occupy a significant percentage of the nanoparticle surface that significantly reduces the amount of MPA molecules bound on the surface. Considering that the intermolecular distance of PEG is 5.5 nm (i.e., the minimal distance giving stable mushroom conformation [4]) and the full saturation of the nanoparticle surface by polymer, we calculated that the amount of MPA molecules will be approximately 11times lower than for the system without PEG. This very nicely corresponds to the intensity drop of the band at 935 cm^{-1} in the blank signals of MPA–NPs and MPA–PEG–NPs (Figure S5), respectively.

REFERENCES

1. Leopold, L.; Todor, I.-S.; Diaconeasa, Z.; Rugină, D.; Ştefancu, A.; Leopold, N.; Coman, C. Assessment of PEG and BSA-PEG gold nanoparticles cellular interaction. *Colloids and Surfaces A: Physicochemical and Engineering Aspects* **2017**, *532*.
2. Lévy, R.; Thanh, N.T.K.; Doty, R.C.; Hussain, I.; Nichols, R.J.; Schiffrin, D.J.; Brust, M.; Fernig, D.G. Rational and Combinatorial Design of Peptide Capping Ligands for Gold Nanoparticles. *J. Am. Chem. Soc.* **2004**, *126*, 10076–10084.
3. Rahme, K.; Chen, L.; Hobbs, R.G.; Morris, M.A.; O'Driscoll, C.; Holmes, J.D. PEGylated gold nanoparticles: polymer quantification as a function of polymer lengths and nanoparticle dimensions. *RSC Adv.* **2013**, *17*, 6085–6094.
4. Labouta, H.I.; Gomez-Garcia, M.J.; Sarsons, C.D.; Nguyen, T.; Kennard, J.; Ngo, W.; Terefe, K.; Iragorri, N.; Lai, P.; Rinker, K.D.; et al. Surface-grafted polyethylene glycol conformation impacts the transport of PEG-functionalized liposomes through a tumour extracellular matrix model. *RSC Adv.* **2018**, *8*, 7697–7708.
5. Castro, J.L.; Lopez-Ramirez M.R.; Arenasand J.F.; Otero J.C. Surface-enhanced Raman scatteringof 3-mercaptopropionic acid adsorbed on a colloidal silver surface. *J. Raman Spectrosc.* **2004**, *35*, 997–1000.
